# Ethical issues in Nipah virus control and research: addressing a neglected disease

**DOI:** 10.1136/jme-2023-109469

**Published:** 2023-12-09

**Authors:** Tess Johnson, Euzebiusz Jamrozik, Tara Hurst, Phaik Yeong Cheah, Michael J Parker

**Affiliations:** 1 Ethox Centre, Nuffield Department of Population Health, University of Oxford, Oxford, UK; 2 Pandemic Sciences Institute, Nuffield Department of Medicine, University of Oxford, Oxford, UK; 3 Royal Melbourne Hospital Department of Medicine, University of Melbourne, Melbourne, Victoria, Australia; 4 Centre for Tropical Medicine and Global Health, Nuffield Department of Medicine, University of Oxford, Oxford, UK; 5 Mahidol-Oxford Tropical Medicine Research, Mahidol University, Bangkok, Thailand

**Keywords:** Communicable Diseases, Ethics- Research, Ethics- Medical, Clinical Trial

## Abstract

Nipah virus is a priority pathogen that is receiving increasing attention among scientists and in work on epidemic preparedness. Despite this trend, there has been almost no bioethical work examining ethical considerations surrounding the epidemiology, prevention, and treatment of Nipah virus or research that has already begun into animal and human vaccines. In this paper, we advance the case for further work on Nipah virus disease in public health ethics due to the distinct issues it raises concerning communication about the modes of transmission, the burdens of public health surveillance, the recent use of stringent public health measures during epidemics, and social or religious norms intersecting with preventive measures. We also advance the case for further work on Nipah virus disease in research ethics, given ethical issues surrounding potential vaccine trials for a high-fatality disease with sporadic spillover events, the different local contexts where trials may occur, and the potential use of unproven therapeutics during outbreaks. Further bioethics work may help to ensure that research and public health interventions for Nipah virus disease are ethically acceptable and more likely to be effective.

## Introduction

**Figure 1:**
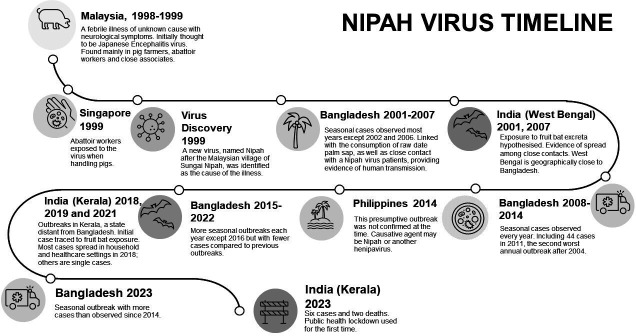
. Timeline of Nipah virus outbreaks.

Nipah virus is a paramyxovirus of the genus Henipavirus. Henipaviruses are primarily carried by fruit bats, causing infections in humans but no signs of illness in bats.^1^ Nipah virus disease was first recognised in humans 25 years ago during outbreaks in Malaysia and Singapore in 1998–1999^1^ (see figure 1). The virus was spread to humans via interaction with pigs, an intermediate host for the virus.^2^ More recently, seasonal outbreaks have occurred in Bangladesh and India due to consumption of fruit and raw date palm sap into which bats have shed virus, although infected bat excreta do not always cause disease.^3^ It remains possible that Nipah could spillover into humans in other countries where fruit bats are found. Much is still unknown about the virus, including the accuracy of reported case rates. Since the original outbreaks, cases in Bangladesh were the first to demonstrate the possibility of human-to-human spread,^1^ primarily via saliva in circumstances of close contact and crowded conditions.^1 4^ The illness may be mild, but can cause encephalitis, coma and death.^5^ Overall, the case fatality rate was around 40% in the original outbreaks in 1998 but has been steady at 70%–75% in Bangladesh.^1 5^

Human-to-human transmission, along with the lack of medical countermeasures and the high fatality rate, has secured Nipah virus a place on the World Health Organization’s priority pathogens list.^6^ It has garnered more attention since the COVID-19 pandemic as global leaders focus on preparing for Disease X. Nipah is considered a possible candidate for another global public health emergency and there are multiple initiatives to develop medical countermeasures to prevent or treat infection. For instance, the Coalition for Epidemic Preparedness has shown increasing interest in tackling the gaps in knowledge and developing medical countermeasures for Nipah, including a greater understanding of the biology and sequence diversity of the virus,^7^ through funding vaccines and treatments. 8 9

In what follows, we argue that further ethical work ought to be done on Nipah virus disease. We consider why Nipah might be neglected and why it should not be, before outlining areas of inquiry in public health ethics and research ethics.

## Why Nipah is neglected and why it should not be

Both public health policies for controlling outbreaks and research regarding Nipah raise numerous ethical issues, for example regarding: (1) the development of ethically acceptable interventions to respond to or prevent outbreaks; (2) delineation of the moral responsibilities of researchers, policy-makers and practitioners in relation to Nipah; (3) incorporation of the values and perspectives of community members who may participate in research and public health interventions; and (4) optimal approaches to Nipah virus research. Yet, despite a wealth of ethical questions surrounding Nipah, to date, there is little published research outside of epidemiology,^1^ and even less specifically featuring ethical analysis of policies, responses, research or outbreak conditions relating to Nipah virus.^2^ There are a number of reasons why this literature may be underdeveloped in comparison to the risk Nipah virus may pose as a priority pathogen.^6^

First, the clearest potential reason might be that Nipah virus is—wrongly, in our view—judged not to raise any unique ethical concerns over and above other priority pathogens such as SARS-CoV-2, Ebola, Lassa fever and Zika. In that case, it may not be seen to require separate ethical analysis. There has been significant research effort directed at issues such as ethical allocation of vaccines in the COVID-19 context,^10 11^ the ethical conduct of clinical research trials during Ebola outbreaks,^9^ injustices such as stigmatisation associated with Lassa fever,^12^ public engagement and involvement in policy responses to Zika virus,^13 14^ and cross-cutting issues such as ethical considerations related to public health surveillance, treatment access, and intersections with existing health disparities.^13 15^ Nipah virus disease has received less attention, despite its importance as a priority pathogen, and despite having several features that are distinct from those of other priority pathogens. These distinct features raise ethical issues especially salient to Nipah. For instance, Nipah virus typically spreads from bats in specific contexts and, to date, human-to-human transmission has been relatively limited, meaning more questions are raised about preventing animal-human spillover events. In addition, with a particularly high case fatality rate and low transmission, we cannot assume that measures used in response to other pathogens with lower case fatality rate and higher transmission will be effective and/or ethically appropriate in the Nipah virus case. Further, methods of Nipah virus transmission from bats to humans and potential mitigation measures may raise concerns regarding respect for traditional practices. In Bangladesh, transmission has been associated with the collection of date palm sap as a traditional delicacy.^1 3^ Mitigation measures that do not consider the importance of these practices for communities and the potential to implement existing, local prevention methods^16^ may be unacceptable or infeasible, as we discuss in detail below.

A second reason why Nipah is neglected in academic literature may be that, even if it does raise new concerns, the attention it has received may be proportionate to the harm it has caused to date. Nipah virus has thus far caused very few deaths compared with mortality from other priority pathogens, with the number of cases ranging from 0 to 67 reported in Bangladesh in 2001–2023.^17^ However, Nipah should arguably receive greater attention and resources insofar as health justice considerations favour the prioritisation of diseases that primarily affect disadvantaged communities.^18^ Moreover, disease burdens associated with Nipah virus may increase in future, for example, because of increased transmissibility, changed climate conditions, increasing natural destruction and intensive land-use in endemic regions,^19^ or acts of bioterrorism.^20 21^

Finally, Nipah may be neglected because there are simply too many higher priorities. More specifically, there may be more pressing health concerns for communities at risk of Nipah exposure, and/or for bioethics researchers. Consider communities first. Issues such as access to stable employment, better health infrastructure, or food security may be more pressing than the risks of Nipah virus for rural populations in the South East Asian countries where Nipah virus outbreaks have occurred to date, especially given that previous Nipah epidemics have been curtailed without extreme public health measures^22^. However, this highlights why there is a particular need for justice considerations to be included in analyses of actions against Nipah virus. There are intersecting vulnerabilities to Nipah virus disease, including those related to employment insecurity^23^ and the effects of climate change.^24^ What is more, communities may have difficulty accessing local healthcare,^25^ and the effects of land exploitation and destruction may play a role in multiple disadvantages.^26^ Recognising the links between risks of Nipah virus exposure and structural conditions can both help in better addressing disease vulnerabilities, and bringing communities’ other needs and priorities surrounding these structural conditions into the spotlight for national and international governance. Next, consider bioethics researchers who may have competing global health research priorities. The COVID-19 pandemic demonstrated how public health emergencies can affect research prioritisation according to salience of the threat.^27 28^ Between outbreaks, there may be little motivation to conduct or fund research on Nipah virus disease. However, we ought to avoid past mistakes: insofar as Nipah is a pathogen with major epidemic or perhaps even pandemic potential^6 20^, this raises justice concerns,^29^ in that Nipah may deserve a higher-priority ranking. Failure to address the threat may result in lack of preparation and much worse outcomes in future outbreaks. Another reason why Nipah virus should be a research priority for bioethicists is its usefulness as a case study for future zoonotic spillover events in general. Since Nipah virus has to date resulted in limited human-to-human transmission, a significant proportion of the (limited) scientific literature on Nipah virus has primarily examined interventions in animals and the environment, to prevent spillover events.^16 24 30^ Nipah virus may be a good candidate for developing research regarding interventions for zoonotic spillover more generally.^31 32^ Ethical analysis may contribute to research regarding causes of zoonotic spillover, and the development of effective and ethically acceptable interventions to change land-use, reduce high-risk contact with wildlife reservoirs and alter human behaviours that act as drivers of spillover.

In the ‘Public health ethics topics’ and ‘Research ethics topics’ sections below, we outline key areas or topics requiring further bioethical work.

## Public health ethics topics

### Transmission and public health communication

One key area for further development within public health ethics is ethically acceptable communication and education on measures to control or prevent Nipah virus transmission. For example, social norms in Bangladesh dictate that family members provide care and are in close contact with infected relatives,^33^ including those infected with Nipah virus. This can exacerbate transmission, and has done so in previous outbreaks. An obvious solution might be the discouragement of close care practices, or isolation, which has been suggested.^34^ However, the biomedical explanation of Nipah virus transmission does not always align with community understanding and advising family members not to care for their relatives may conflict with key values. In an anthropological case study on Nipah,^33^ members of one village associated Nipah virus disease with people spending too long in the sun, with punishment from Allah, and with a supernatural force. The latter explanation was reinforced by the lack of effective treatment for those suffering from Nipah virus symptoms. Furthermore, interviewees were resistant to the idea of human-to-human spread through close contact, noting the rarity of such transmission and numerous examples where family members had had close contact with an infected individual without themselves becoming infected. This study shows how much more work is needed examining the design of ethical infection control and public health communication measures and how this may be influenced by social norms and religious requirements relating to Nipah. It may be that such measures can be designed to work around social norms and religious requirements.^16^ If this is not the case, more work will be needed to ensure that public health communication is presented with adequate evidence, relevance and involvement of communities. This is emphasised by previous cases of community mistrust in doctors advising preventive measures against Nipah virus, where doctors explained the cause of Nipah as consuming raw date palm sap without explaining that this was because the sap was often contaminated by bats.^35^ Research aiming to improve the local ethical acceptability of prevention measures can also lead to co-creation of measures, as with the development of bamboo skirts for date palm sap pipes and jugs developed with sap harvesters in Bangladesh.^16^

### Stigmatisation and politicisation

A second area for work in public health ethics on Nipah virus is exploring the potential for stigmatisation and politicisation of control and prevention measures. An example from Malaysia can be used to highlight this possible ethical issue. In Malaysian outbreaks, Nipah transmission to humans has occurred via contact with pigs. This means that the populations most likely to be infected are farmers who raise pigs, those administering injections to pigs, those assisting in the birth of piglets, and their local communities. Since the consumption of pork is forbidden among the majority Muslim population of Malaysia, pig farmers are more likely to be among the non-Muslim minority. While the raising and selling of pigs is one contention, the potential for the pigs to carry and spread diseases to the Malaysian population has been a further locus of social tensions and can result in further stigma for the minority non-Muslim pig-farming population. The proliferation of pig farms in Malaysia was noted by the United Nations Development Programme as causing ‘religious and social problems’ as far back as 1975.^36^ Various regulatory changes led to the semilegal ‘occupation’ of pig farms in one state of Malaysia in the early 1990s^37^, and when Nipah virus outbreaks occurred in farms in other states of Malaysia, it was suggested that all pig farms in the state should be shut down. Pig farmers responded with protests and tensions between the Muslim and non-Muslim population arose. The industry as a whole remains destabilised and politicised. This situation might be exacerbated further by an association between farmers and Nipah virus in the event of future significant outbreaks. Preventive measures such as pig culls and animal vaccination ought to be considered as situated in the context of a politically and socially tense, destabilised industry.

### Surveillance burdening disadvantaged populations

A key public health measure to monitor and control Nipah virus outbreaks is the ongoing development of public health surveillance systems. By detecting cases early in humans (and/or animal reservoirs), surveillance may help to focus measures that prevent the spread of infection where risk is highest—and may, in the long term, increase scientific knowledge regarding the epidemiology of Nipah. Surveillance for Nipah raises a range of ethical issues, in particular because—as for several other pathogens—the populations most likely to be subject to surveillance and resultant public health interventions are often poor and rural communities with multiple intersecting vulnerabilities, who are then further burdened with prevention measures.^15^ While local communities may, in principle, benefit from surveillance and the early detection of outbreaks, data sharing with distant government agencies and public health interventions may not always be welcomed (especially if designed without adequate community consultation).^34^ Further, one reason for surveillance is the prevention of spread of disease to other populations (e.g., capital cities or international centres) who stand to benefit from such public health activities while the burdens of these activities are concentrated in high-risk communities.^15^ Such asymmetries in the distribution of risk as well as the benefits and burdens of public health activities arguably give rise to ethical obligations for public health agencies. First, the principle of reciprocity requires that those who are burdened by public health activities should receive adequate assistance (e.g., access to Nipah virus testing and care) and compensation (e.g., for costs arising from public health interventions).^38^ It is reasonable to expect national and international funding for such reciprocal support, given the wider benefits of preventing the spread of Nipah virus. Second, there is arguably an ethical obligation of community engagement to inform the design and conduct of public health activities. These should be sensitive to local priorities and values as a matter of respect for affected populations, but also because context-sensitive public health programmes may produce more benefits and fewer harms.

### Use of excessively stringent public health measures

In mid-September 2023, two deaths were registered from Nipah virus disease in a hospital in Kerala, India. The regional government subsequently created seven containment zones where business activity was limited, schools were closed, and social distancing and mask mandates were put in place.^39^ In addition, neighbouring Indian states have been put on alert,^39^ and arrivals from India to locations such as Bali, Indonesia, face screening intended to detect potential Nipah cases.^40^ These policies seem to mirror those often implemented during the COVID-19 pandemic.

It is important to avoid unreflectively applying control measures from the pandemic context to the case of Nipah virus disease. For example, Nipah virus has different transmission dynamics to those observed in respiratory virus pandemics such as COVID-19. On the one hand, Nipah is less transmissible than pandemic respiratory viruses, with the majority of human-to-human spread occuring among household members and patients or staff exposed in healthcare settings^22^. This renders community interventions commonly used against respiratory diseases less effective (e.g., because general community measures do not reduce household and healthcare spread of infection) and therefore less ethically justifiable. On the other hand, Nipah is also more fatal than average pandemic respiratory viruses, meaning that each case of transmission prevented may avoid a large potential harm to individuals, however this does not necessarily mean that extreme measures to prevent Nipah virus spread are ethically justifiable (e.g., cordons sanitaires, lockdowns, etc.). Border screening also lacks a strong ethical basis given that it is unlikely to be be sensitive or specific enough to be efficient for the detection of rare infected individuals. Moreover, a high fatality and significant transmission risk to close contacts means that stay-at-home orders and household quarantine (if not carefully implemented) might plausibly *increase* risks of transmission within households during outbreaks.

Social distancing, lockdown, travel restrictions, and border screening are therefore unlikely to reduce transmission of Nipah to a significant degree, while being likely to cause harms including lost livelihoods, increased risk of domestic abuse, adverse effects on mental health, potential increased spread of infection in some contexts, and the undermining of public trust. Further, it is now well recognised that the harms of stringent public health measures are often concentrated among disadvantaged groups.^41^ If the transmission dynamics of Nipah virus were to significantly change toward higher risk of human-to-human spread among casual contacts, wider public health measures may, under certain conditions, become more ethically acceptable. However, such measures are not currently justifable given that most spread of Nipah virus occurs among close contacts, and the previous Kerala outbreak in 2018 was controlled without extreme measures such as lockdown - with the response instead focusing on healthcare settings in particular 22.

Public health agencies should therefore avoid assumptions that measures used for COVID19 (or other epidemics with different features) would be effective or ethically justifiable for Nipah outbreaks. In general, less restrictive measures should be preferred to more restrictive alternatives. For example, standard measures such as contact tracing may be very effective against Nipah virus transmission, under the current transmission dynamics, provided that affected communities trust public health agencies enough to participate - and excessively restrictive measures may undermine trust. While common public health ethics principles and frameworks^38 42^ may be applicable, Nipah outbreak epidemic responses may yet face new challenges and raise new ethical questions, given different biomedical and contextual factors as compared with other epidemics.

## Research ethics topics

Currently there are vaccines in development for Nipah virus. However, conducting vaccine trials is challenging due to the low incidence and sporadic nature of disease outbreaks. After initial first-in-human trials in a small group of people in highly controlled settings, phase 2 clinical trials would most likely be conducted in non-outbreak settings with healthy volunteers, in whom T cell activation or the production of neutralising antibodies can be used as evidence of protective immune response.^43^ This is necessary to ensure that the vaccines are ready for roll-out during an outbreak, where it would be ethically questionable to use a control arm, whether placebo or another vaccine.

### Phases 1 and 2 vaccine trials

Early phase vaccine trials in healthy volunteers are essential to provide data on safety (phase 1) and immunogenicity (phase 2) prior to an outbreak. Ideally, these trials should take place in locations with populations where a roll-out is likely, such as populations that harvest date palm sap in Bangladesh and India, and pig farmers in Malaysia. Among other things, conducting phase 1 and 2 trials in individuals at high risk of future exposure provides the opportunity to enrol the same individuals in any subsequent phase 3 efficacy trials. However, trials in these settings pose numerous challenges. There is a low level of scientific and health literacy, and communities hold different beliefs about the origins of disease,^35^ which may make the informed consent process challenging. Moreover, understanding the reasons for vaccine trials in the absence of an outbreak can be difficult. Vaccine trials could potentially create anxieties among the population, as they might wonder whether there is an actual outbreak that is not being revealed to them. There is also a potential for stigmatising trial populations. In Malaysia, pig farmers were thought to have spread the Nipah virus after catching it from infected pigs.^2 37^ If they were to take part in these trials, they might face further stigmatisation.

### Phase 3 vaccine trials

The design of vaccine efficacy (phase 3) trials for Nipah virus disease may also raise several practical and ethical challenges.^44^ Demonstrating efficacy in humans in a phase 3 vaccine trial can be difficult because of the sporadic nature of spillover events and the fact that suppression of transmission via public health measures, while beneficial, may reduce the probability that vaccine trial participants are infected (which is required for the trial to prove that vaccines are efficacious). Vaccine trials may, therefore, be likely to recruit healthcare workers in high-risk areas (either before or during epidemics) and/or community members who are likely to have contact with bats, pigs or infectious human cases (e.g., household contacts of cases). One option, used for Ebola vaccine trials, would be a ring vaccination design, where the (‘ring’ of) contacts of infectious cases are randomised to receive an experimental vaccine or placebo.^45^ However, such study designs may be highly infeasible in the current Nipah context.^44^ In any case, the high mortality of Nipah virus and the perception or presumption that an experimental vaccine is more likely to provide benefit than a placebo (although this presumption is not always reliable) may influence on trial design, including where local communities would not accept a placebo arm. There should ideally be prospective community engagement to determine whether trials using a control arm with placebo (or a licensed vaccine for a disease other than Nipah) are acceptable, given that this would be the most reliable method of determining vaccine efficacy. If local populations do not accept standard placebo designs due to concerns regarding risks of disease in the placebo arm, other designs might be tried. These might include stepped wedge randomisation or immediate *vs* delayed vaccination which may result in all trial participants being vaccinated without unduly undermining analyses of vaccine efficacy.^44 45^

### Therapeutics and non-pharmaceutical interventions

Trials of therapeutics and non-pharmaceutical interventions may also raise ethical issues. Public health emergencies are often associated with use of unproven interventions,^46 47^ sometimes out of a perception that potential benefits outweigh potential harms. As far as possible, there is arguably an ethical imperative that unproven interventions should be tested in rigorous trial designs rather than in ad hoc clinical or public health use, as this is the most expedient way to produce evidence to guide practice—as illustrated by trials of COVID-19 therapeutics.^47^ Where rigorous trials are not acceptable to local populations, other approaches such as the monitored emergency use of unregistered interventions framework^48^ may help to maximise the production of reliable data (even if these data may not be as reliable as those from formal clinical trials). Similarly, there is an ethical imperative to collect data regarding the benefits and harms of non-pharmaceutical interventions, as this may help to refine public health control strategies over time by improving the balance of benefits and harms as well as determining which measures are most acceptable to local communities. More broadly, there is increasing awareness of the ethical case for trials of policy,^49^ and the rationale for such research may be particularly compelling for high-consequence pathogens such as Nipah virus. In addition to the kinds of research described above, social and behavioural research is increasingly recognised as vital to effective epidemic prevention, preparedness and response. For example, social and behavioural research may help to improve the design and conduct of intervention trials as well as public health programmes. Yet social and behavioural research itself may also raise specific ethical issues which should be identified, analysed and addressed.

## Conclusion

In this paper, we have argued that Nipah virus disease should receive more attention in the bioethics literature. There are several distinctive characteristics of Nipah virus that raise important ethical questions, many of which are not present for other priority pathogens. Furthermore, the disease burden may increase significantly in future, and the health injustice concerns raised already indicate it already deserves further ethical attention.

In public health ethics, further bioethics research ought to focus on stigmatisation and politicisation of preventive measures, especially surrounding pig culls, effective and respectful public health communication, the burden associated with public health surveillance of already-disadvantaged populations, and avoiding inappropriate transplantation of control measures designed for other disease contexts. In research ethics, research ought to focus on effective vaccine trial design in the context of a disease with distinctively sporadic zoonotic spillover. It ought to also focus on the adaptation of vaccine trials to specific local contexts where possible and necessary, on health policy trials, and on reducing the use of unproven therapeutics.

A key feature of work going forward must be collaboration with local bioethicists, biomedical scientists, and communities (likely to be) experiencing Nipah virus outbreaks, to ensure that the development of interventions considers all ethically relevant factors. Nipah virus is now a priority pathogen. The threats it poses to global health and health equity, as well as the need to develop ethically appropriate research and public health responses, are significant reasons for more work, including in bioethics, to address this previously neglected disaese.

## Data Availability

Data sharing not applicable as no datasets generated and/or analysed for this study.

## Footnotes

Although, for exceptions, see papers examining policy and human causal transmission factors.^31 32 49^

Although, for an exception, see one paper on ethical considerations in deploying healthcare workers in Nipah-affected areas.^13^
